# Prolonged CBD Exposure Is Associated with Higher Expression of *flgA* in *Salmonella* Typhimurium

**DOI:** 10.3390/antibiotics15100945

**Published:** 2026-09-23

**Authors:** Iddrisu Ibrahim, Alexa Mathias, Daron Johnson, Kelci Lawrence, Othreniel Angel Forte, Navitri Chandra Naidu, Latrell Huitt, Nathaniel Ajibola, Junhuan Xu, Robertson K. Boakai, Fortune Akabanda, James Owusu-Kwarteng, Olufemi S. Ajayi, Komal Vig, Joseph Atia Ayariga

**Affiliations:** 1 The Microbiology Program, College of Science, Technology, Engineering, and Mathematics (C-STEM), Alabama State University, Montgomery, AL 36104, USA; iiddrisu3715@alasu.edu (I.I.); djohnson@alasu.edu (D.J.); klawrence0670@alasu.edu (K.L.); oforte3773@myasu.alasu.edu (O.A.F.); nnaidu@alasu.edu (N.C.N.); lhuitt@alasu.edu (L.H.); najibola7327@alasu.edu (N.A.); jxu@alasu.edu (J.X.); brobertson@alasu.edu (R.K.B.); komalvig@alasu.edu (K.V.); 2 The Industrial Hemp Program, College of Science, Technology, Engineering, and Mathematics (C-STEM), Alabama State University, Montgomery, AL 36104, USA; 3 African Drug Discovery and Innovation Network, Sunyani P.O. Box AB45, Ghana; james.owusu-kwarteng@uenr.edu.gh; 4 Department of Food Science and Technology, University for Development Studies, Tamale P.O. Box TL1350, Ghana; fakabanda@uds.edu.gh; 5 Department of Food Science and Technology, School of Agriculture and Technology, University of Energy and Natural Resources, Sunyani P.O. Box 214, Ghana

**Keywords:** bacterial motility, bacterial survival, cannabidiol (CBD), CBD adaptation, environmental stress, *Salmonella* Typhimurium

## Abstract

Background/Objectives: *Salmonella* Typhimurium is a Gram-negative foodborne pathogen that remains a major public-health concern. In this study, we examined whether prolonged exposure to cannabidiol (CBD) was associated with altered *flgA* expression and related phenotypes in a laboratory-derived CBD-adapted strain compared with its susceptible parental strain. Methods: We assessed relative *flgA* mRNA expression, motility, growth at different pH conditions, responses to nutrient starvation, and biofilm formation. Results: The CBD-adapted strain showed approximately sixfold higher *flgA* mRNA expression and greater motility at pH 7. However, the pH findings were mixed: neither strain showed measurable migration at pH 3, the limited migration observed for the adapted strain at pH 12 was highly variable, and both strains showed reduced growth at extreme pH. Under nutrient starvation, relative OD_600_ declined in both strains, with a faster decline in the CBD-adapted strain under several CBD conditions. Biofilm measurements also differed by strain, CBD concentration, and incubation time. Conclusions: These findings show an association between prolonged CBD exposure, higher *flgA* expression, and altered phenotypes, but they do not establish that *flgA* caused the observed changes. The study therefore provides an exploratory basis for targeted genetic and phenotypic validation of CBD adaptation in *Salmonella* Typhimurium. Because the repeated measurements were technical replicates rather than independent biological replicates, the findings are descriptive and limited to the two strains examined.

## 1. Introduction

*Salmonella* Typhimurium is a foodborne pathogen of public-health importance. In the United States, *Salmonella* infections collectively—not *Salmonella* Typhimurium alone—are estimated to cause approximately 1.35 million illnesses, 26,500 hospitalizations, and 420 deaths each year [1,2]. More than 2500 *Salmonella* serotypes have been described, and *Salmonella* Typhimurium is among the serotypes frequently associated with human and animal infections. Its virulence factors support adhesion, invasion, intracellular survival, and persistence in the host [2,3]. One of these factors is the flagellum, a dynamic structure that supports motility and environmental sensing. A typical bacterial flagellum comprises a filament, hook, and basal body that work together to propel the cell. Rotation of the motor is powered by an electrochemical ion gradient, usually the proton motive force in *Salmonella*, rather than by the basal body “driving energy” through the membrane [4]. The P ring stabilizes the flagellar rod within the peptidoglycan layer. FlgI is the structural P-ring protein, whereas FlgA is a periplasmic chaperone that supports FlgI folding and P-ring assembly [4,5].

Flagellar gene expression is organized in a regulatory hierarchy. The class I *flhDC* operon encodes the FlhD4C2 master regulator, which activates class II genes required for the hook–basal-body and export apparatus. Class II activity then permits expression of class III genes involved in the filament, motor function, and chemotaxis. The *flgA* and *flgI* genes belong to the assembly program: *flgA* encodes the periplasmic chaperone FlgA, while *flgI* encodes the P-ring structural protein FlgI [4,5]. It is therefore not accurate to identify one gene as the single “most important” member of the family. FlhD4C2 controls the hierarchy, while FlgA performs a specific and necessary role in P-ring assembly. Their functions are coordinated rather than interchangeable. The coordinated roles of these genes in flagellar assembly are illustrated in Figure 1.

Among the dozens of phytocannabinoids, cannabidiol (CBD), a non-intoxicating constituent of *Cannabis sativa*, has attracted interest because of its reported anxiolytic, anti-inflammatory, and analgesic properties [6]. Unlike delta-9-tetrahydrocannabinol (THC), CBD does not produce the characteristic intoxicating “high,” although its clinical use is not free of risk [7]. Reported adverse effects include diarrhea, somnolence, decreased appetite, fatigue, and elevations in liver transaminases; clinically important drug–drug interactions can also occur [8]. Much of the existing work on CBD has focused on mammalian conditions such as pain, anxiety, epilepsy, and multiple sclerosis. More recently, early-stage studies have examined its antibacterial activity, including activity against *Salmonella* [9,10]. Our laboratory previously explored mechanisms associated with adaptation of *Salmonella* Typhimurium to CBD [11]. Figure 2 summarizes the structural organization of the flagellum and the FlgA-centered functional association network. The objective of the present study was to assess whether prolonged CBD exposure was associated with altered *flgA* expression and with changes in motility, pH response, nutrient-starvation response, and biofilm formation. We hypothesized an association between CBD adaptation and *flgA* upregulation; because *flgA* was not genetically manipulated, we did not treat it as the proven cause of the other phenotypes.

## 2. Results

### 2.1. Motility Assay

A comparative analysis of *flgA* expression showed that the laboratory-derived CBD-adapted strain had approximately sixfold higher relative *flgA* mRNA expression than the CBD-susceptible parental strain of *Salmonella* Typhimurium (Figure 3). This comparison establishes an association with the adapted phenotype; it does not establish that *flgA* upregulation caused the other phenotypic differences. The approximate fold difference describes this assay only because the qPCR measurements were technical replicates.

Motility was assessed alongside *flgA* expression in the laboratory-derived CBD-adapted and susceptible strains. Figure 4 shows no measurable migration for either strain at pH 3. At pH 7, both strains migrated, and the CBD-adapted strain showed a larger migration radius than the susceptible strain. At pH 12, no measurable migration was observed for the susceptible strain, while limited and highly variable migration was observed for the adapted strain. These measurements describe migration on motility agar and should not be interpreted as a direct viability assay.

After 48 h of incubation at pH 3, no measurable migration was observed for either strain. At pH 12, the susceptible strain also showed no measurable migration, whereas the CBD-adapted strain had a reported mean migration radius of 1.6 ± 2.26 cm across the technical replicate plates (Table 1). These values describe within-assay technical variation and were not used for inferential testing. Because the variation was larger than the mean and no confirmatory viability assay was performed, we do not interpret these data as evidence that one strain survived while the other did not.

At pH 7, the laboratory-derived CBD-adapted strain exhibited a larger migration radius than the susceptible strain at the measured time points (Figure 5). We interpret this as an association between the adapted phenotype and increased motility under the tested neutral condition. We do not extend this observation to all pH conditions: both strains showed no measurable migration at pH 3, and the pH 12 result for the adapted strain was highly variable. Any effect on host invasion remains a hypothesis because no invasion or infection assay was performed.

### 2.2. Growth Responses at Different pH Levels

The pH conditions produced different growth patterns in the laboratory-derived CBD-adapted and susceptible strains (Figure 6). At pH 7, both strains increased in relative OD_600_ over time, while at pH 5 both strains also increased and the adapted strain reached a higher percentage change than the susceptible strain. At pH 3 and pH 12, relative OD_600_ declined in both strains. At the end of the 12 h period, the decline was slightly greater in the adapted strain at both extreme pH conditions. The complete dataset therefore does not support a general claim that CBD adaptation improved resilience across all pH values.

### 2.3. Growth Kinetics of CBD-Adapted and CBD-Susceptible S. Typhimurium Under Nutrient Starvation and CBD Exposure

Under nutrient starvation, relative OD_600_ declined in both the laboratory-derived CBD-adapted and susceptible strains across the tested CBD conditions (Figure 7). The susceptible strain showed a small, short-lived increase during the early time points in some conditions, whereas the adapted strain declined earlier. Because OD_600_ does not distinguish viable cells from non-viable cells or cellular debris, these curves are described as changes in optical density rather than direct evidence of bacterial death or CBD utilization.

Figure 8 presents the growth kinetics of the laboratory-derived CBD-adapted and susceptible strains separately under nutrient starvation and the tested CBD concentrations. The susceptible strain maintained a small positive change in OD_600_ during some early time points, whereas the adapted strain showed an earlier decline. These optical-density data do not by themselves establish viability, metabolic use of CBD, or the mechanism responsible for the different curves.

### 2.4. Biofilm Assessment

Scanning electron microscopy showed morphological differences between biofilms produced by the laboratory-derived CBD-adapted and susceptible strains. Under the tested CBD treatments, the adapted strain retained more biofilm-associated material than the susceptible strain (Figure 9). We describe this as a morphological observation under the present conditions; it does not establish that *flgA* caused biofilm formation or that the phenotype increased infection persistence.

For the quantitative biofilm assessment, both strains were examined after 24 h and 60 h under the indicated CBD concentrations (Figure 10). The magnitude and direction of the differences varied by strain, concentration, and time. We therefore present the pattern as time- and concentration-dependent rather than as a uniform increase or decrease in biofilm formation.

The crystal-violet microphotographs indicated that the laboratory-derived CBD-adapted strain retained more biofilm-associated material under 5 µg/mL of CBD than under 100 µg/mL of CBD, while the susceptible strain showed a different concentration-dependent pattern (Figure 11). The stronger reduction observed at the lower concentration and later time point was not interpreted as a simple dose–response relationship. It may reflect a non-monotonic, time-dependent response or technical variation and requires confirmation with independent biological replicates and additional concentrations.

## 3. Discussion

The present study compared *flgA* transcript abundance with motility, pH response, nutrient-starvation response, and biofilm phenotypes in a susceptible parental strain and a laboratory-derived CBD-adapted derivative. FlgA is a periplasmic chaperone required for efficient assembly of FlgI into the P ring; it is not itself the P-ring structural protein [4,5]. The observed co-occurrence of higher *flgA* mRNA and greater motility at pH 7 is biologically plausible, but the study did not manipulate *flgA* and therefore cannot assign causality to this gene.

Our primary aim was to assess how prolonged CBD exposure was associated with *flgA* expression in *Salmonella* Typhimurium. The laboratory-derived CBD-adapted strain showed higher *flgA* mRNA than the susceptible parental strain (Figure 3), together with greater motility at pH 7 (Figure 5). These findings support an association, not proof, that *flgA* upregulation was responsible for adherence, biofilm formation, environmental fitness, survival, or virulence. Zhang et al. reported that disruption of *luxS* in *Vibrio harveyi* altered the expression of several flagellar genes, including *flgA*, and affected motility [12]. Because the organism, regulatory pathway, and experimental design differ from the present study, we use that work only as indirect context. Matsunami et al. demonstrated that structural flexibility of FlgA is important for its chaperone function during P-ring assembly [5]. That mechanistic evidence supports the role of FlgA in flagellar assembly but does not establish that the higher *flgA* expression observed here caused the other phenotypes or enhanced host colonization or immune evasion.

Environmental pH influences bacterial physiology, growth, and expression of stress-response pathways [13,14]. In our motility experiment, neither strain showed measurable migration at pH 3, while the adapted strain showed a small and highly variable migration radius at pH 12 (Figure 4; Table 1). In the liquid-growth experiment, both strains increased in relative OD_600_ at pH 5 and pH 7, whereas both declined at pH 3 and pH 12 (Figure 6). These results show that the effect of pH depended on the assay and condition. They do not demonstrate that pH regulated antibiotic resistance, and they do not establish that a zero-migration measurement represented loss of viability.

The pH-growth results also showed that the laboratory-derived CBD-adapted strain did not consistently outperform the susceptible strain. The adapted strain reached a higher relative OD_600_ change at pH 5, but it declined slightly more at pH 3 and pH 12 by the end of the experiment. This mixed pattern is consistent with the broad dependence of bacterial growth on proton homeostasis and acid- or alkali-stress responses [14,15]. We therefore removed the comparison with *Candida albicans* and *Aloe vera* because it did not directly inform CBD adaptation in *Salmonella*. Nutrient availability can also influence motility and biofilm development by altering cellular metabolism and the resources available for flagellar assembly [16,17].

We also analyzed relative OD_600_ under nutrient starvation and varying CBD concentrations (Figure 7). Both strains declined under the tested conditions, although the susceptible strain showed a small, temporary increase during some early time points. Starvation responses depend on the composition of the medium and the balance between cellular maintenance and available resources [18]. The present study did not measure cellular ATP, proton motive force, efflux activity, CBD catabolism, or viability. We therefore removed the earlier explanation that the adapted strain died because flagellar expression consumed ATP or because the susceptible strain used CBD as an energy source. Our previous work identified increased *ompC* expression in a CBD-adapted strain [11], but OmpC is an outer-membrane porin rather than an efflux pump. Its expression cannot be used as evidence of ATP-dependent CBD export without direct transport experiments.

In the presence of 5 µg/mL of CBD under nutrient starvation, both strains showed a decline in relative OD_600_, while the susceptible strain displayed a small early increase before declining (Figure 7B). Similar strain-dependent patterns occurred at the other CBD concentrations. These findings suggest that the adapted and susceptible strains responded differently to the combined conditions, but they do not identify the underlying mechanism or demonstrate loss of viability.

Figure 8 presents the strain-specific curves across the nutrient-starvation conditions. The susceptible control showed the earliest decline, while some CBD-treated susceptible groups remained above baseline for longer. The adapted strain showed negative relative OD_600_ values across the tested conditions. We have limited our interpretation to these measured optical-density patterns. We do not infer a sixfold increase in energy use from a sixfold difference in *flgA* mRNA, and we do not infer ATP depletion, efflux-pump activity, CBD utilization, or starvation-induced death because those endpoints were not measured.

### Study Limitations and Future Directions

This study used one laboratory-derived CBD-adapted derivative and its susceptible parental strain. The repeated measurements were technical replicates rather than independent biological replicates, so the findings are descriptive and should be interpreted as exploratory. Reanalyzing technical replicates with either a parametric or nonparametric test would not provide biological replication or support generalization beyond the two strains. The original motility design did not include pH 5, and a zero migration radius was not confirmed by CFU or another viability assay. The study also did not determine MIC/MBC values in the present experiment, test stability after passage without CBD, genetically manipulate *flgA*, or directly measure invasion, biofilm causation, ATP, proton motive force, CBD transport, or CBD catabolism. In addition, CBD was present immediately before RNA extraction for the adapted strain, so acute exposure and stable adaptation cannot be separated completely. Future work should use independent biological cultures and several independently derived adapted lineages; include additional isolates; determine MIC, MBC, and resistance stability; include pH 5 in both motility and growth assays; use CFU-based viability measurements; and compare wild-type, *flgA*-deficient, complemented, and controlled-overexpression strains. Inferential testing should be performed only after the experimental unit and independent biological replication have been defined.

## 4. Materials and Methods

### 4.1. Media, Chemicals, Bacterial Strains, and Other Reagents

All chemicals used in this study—including hexane, LC–MS-grade methanol (≥99.9%), water, and absolute ethanol (≥99.5%)—were of HPLC grade. The parental strain was the laboratory-coded *Salmonella* Typhimurium BV4012 strain, derived from *Salmonella* Typhimurium LT2 strain MS1868. The present work compared that CBD-susceptible parental strain with one laboratory-derived CBD-adapted derivative. As reported in our earlier work, BV4012 was exposed to CBD-containing medium for 7 days, beginning at 1–5 µg/mL; colonies were then selected and propagated at 10–50 µg/mL of CBD, and the final selected colonies were plated on LB agar containing 50 µg/mL of CBD [10]. The adapted derivative was routinely maintained in LB containing at least 10 µg/mL of CBD. “CBD-adapted” is used here as an operational description of this selection history rather than as a substitute for a newly determined MIC or MBC in the present study. Luria broth and Luria agar (BD Difco, Franklin Lakes, NJ, USA) were used for routine maintenance. The CBD extract was supplied by Sustainable CBD LLC (Salem, AL, USA), and its extraction and purification were described previously [9].

### 4.2. RNA Extraction

The protocol provided with the RNeasy Kit (QIAGEN Sciences LLC, Germantown, MD, USA) was used to extract RNA from both CBD-susceptible and CBD-adapted strains of *Salmonella*. Extracted RNA samples were treated with DNase I (Thermo Scientific™, Vilnius, Lithuania) to remove contaminating genomic DNA. Following DNase-I treatment, the concentration and purity of the RNA were determined using a NanoDrop 1000 spectrophotometer (Thermo Fisher Scientific, Madison, WI, USA).

### 4.3. Reverse Transcription

Complementary DNA (cDNA) was synthesized from purified total RNA using the SuperScript™ IV Reverse Transcriptase system (Invitrogen, Thermo Fisher Scientific, Vilnius, Lithuania). Prior to reverse transcription, RNA concentrations were normalized to ensure that an equivalent amount of RNA was used for each reaction. A total of 1 µg of RNA was used as the template for each cDNA synthesis reaction.

For primer annealing, 1 µg of total RNA was combined with 1 µL of dNTP mix (10 mM each) and 1 µL of 50 µM random primer mixture. Nuclease-free water was added to bring the total volume to 13 µL. The RNA–primer mixture was gently mixed and incubated at 65 °C for 5 min to facilitate RNA denaturation and primer accessibility, followed by immediate incubation on ice for 1 min.

The reverse transcription reaction was subsequently assembled by adding 4 µL of 5× SuperScript™ IV reaction buffer, 1 µL of 100 mM dithiothreitol (DTT), 1 µL of RNaseOUT™ Recombinant Ribonuclease Inhibitor (40 U/µL), and 1 µL of SuperScript™ IV Reverse Transcriptase (200 U/µL; Lot No. 00916637; Invitrogen, Thermo Fisher Scientific, Vilnius, Lithuania) to the 13-µL RNA–primer mixture. This resulted in a final reaction volume of 20 µL containing 1 µg of total RNA.

Reverse transcription was performed by incubating the reaction mixture at 50 °C for 10 min. The reverse transcriptase was subsequently heat-inactivated at 80 °C for 10 min. Following completion of the reaction, the synthesized cDNA was briefly cooled and stored at −20 °C until subsequent downstream analysis. To minimize RNA degradation and experimental variability, all RNA samples were handled using RNase-free reagents, tubes, and pipette tips, and samples were maintained on ice whenever appropriate during reaction preparation.

### 4.4. Quantitative PCR Analysis of Gene Expression

Quantitative real-time PCR (qPCR) was performed to determine the relative expression of the flagellar biosynthesis gene *flgA* in laboratory-derived cannabidiol (CBD)-adapted and CBD-susceptible parental *Salmonella enterica* serovar Typhimurium strains. The three repeated qPCR measurements for each strain were technical replicates, not independent biological replicates. Prior to RNA extraction, both strains were cultured overnight in Luria–Bertani (LB) broth at pH 7.0 under their respective maintenance conditions. The CBD-adapted strain was cultured in the presence of 10 µg/mL of CBD, whereas the CBD-susceptible parental strain was cultured under identical conditions without CBD. Total RNA was extracted from each strain preparation, resuspended in RNase-free water, and quantified using a NanoDrop 1000 spectrophotometer (Thermo Fisher Scientific). RNA samples were normalized prior to reverse transcription, and 1 µg of total RNA from each sample was converted to complementary DNA (cDNA) using SuperScript™ IV Reverse Transcriptase as described in Section 4.3. The resulting cDNA served as the template for qPCR analysis.

Expression of *flgA* was quantified using the following gene-specific primers: *flgA* forward, 5′-TTCATCGCCCAATCTGCTAC-3′, and *flgA* reverse, 5′-GACGGTTCCACTCACGATTT-3′. DNA gyrase subunit B (*gyrB*) was used as the endogenous reference gene for normalization. The *gyrB* primers were forward, 5′-GTTGGTGAAGGTTTCGTGGC-3′, and reverse, 5′-ATATCGGCGACACGGATGAC-3′.

qPCR reactions were performed using a SYBR Green-based master mix (lot# 00914521, Thermo Fisher Scientific) on a CFX96 Real-Time PCR Detection System (Bio-Rad Laboratories, Hercules, CA, USA). Each reaction was performed in a final volume of 20 µL, containing 5 µL of 2× qPCR master mix, 1 µL each of the forward and reverse primers (0.5 µM final concentration each), 1 µL of cDNA template, and nuclease-free water to the final reaction volume.

Each strain sample was analyzed in technical triplicate for both *flgA* and *gyrB*. Technical replicate Ct values were examined for consistency and averaged within the sample before descriptive relative-expression calculations. Amplification was performed using the following thermal cycling program: an initial enzyme denaturation step at 95 °C for 3 min, followed by 45 cycles of denaturation at 95 °C for 30 s, primer annealing at 65 °C for 30 s, and extension at 72 °C for 30 s. Fluorescence data were acquired during the amplification phase according to the settings of the CFX96 system.

Following amplification, a melting-curve analysis was performed to evaluate amplification specificity. The temperature was increased progressively from 65 °C to 95 °C, with fluorescence monitored throughout the temperature ramp. A single, well-defined melting peak was required for each primer pair and was interpreted as evidence of a predominant specific amplification product. Reactions displaying nonspecific melting profiles or multiple peaks were excluded or repeated. Prior to relative gene-expression analysis, the amplification performance of the *flgA* and *gyrB* primer pairs was evaluated using serial dilutions of cDNA. Standard curves were generated by plotting Ct values against the logarithm of template concentration. Amplification efficiencies were calculated from the slopes of the respective standard curves. Primer pairs were considered suitable for relative-expression analysis when amplification efficiencies were within an acceptable range and were sufficiently comparable between the target (*flgA*) and reference (*gyrB*) assays.

Appropriate negative controls were included in each qPCR experiment. No-template controls (NTCs), in which nuclease-free water replaced the cDNA template, were included for each primer pair to detect reagent contamination or nonspecific amplification. Where performed, no-reverse-transcriptase (−RT) controls were included to assess potential amplification arising from residual genomic DNA. Negative controls were required to show no detectable specific amplification within the analytical Ct range. Melting-curve profiles were additionally examined to distinguish specific amplification from nonspecific products or primer-dimer formation. Relative *flgA* expression was determined by normalizing the Ct value of *flgA* to that of the endogenous reference gene *gyrB*. For each strain sample, ΔCt was calculated as the difference between the Ct values of *flgA* and *gyrB*. The CBD-susceptible parental strain served as the calibrator, and the difference between the CBD-adapted and susceptible strains was expressed as a descriptive relative fold change using the 2^−ΔΔCt^ method, provided that the experimentally determined amplification efficiencies of the target and reference assays were sufficiently similar to support this approach. The averaged technical-replicate Ct values produced one descriptive result per strain; they were not treated as independent observations, and no inferential statistical test was applied.

### 4.5. Motility Assessment at Different pH Levels

The effect of environmental pH on the motility of CBD-adapted and CBD-susceptible *Salmonella enterica* serovar Typhimurium strains was evaluated using a semi-solid agar motility assay, following the general approach described by Palma et al. [19], with modifications. Motility assays were performed using medium containing 0.3% (*w*/*v*) agar, which provided a semi-solid matrix suitable for assessing bacterial migration away from the point of inoculation. For the different pH treatments, the motility medium was adjusted to pH 3.0, 7.0, or 12.0. Acidic conditions were established by gradually adding 1 N HCl, whereas alkaline conditions were established using 1 N NaOH, with continuous stirring. The pH was monitored during adjustment and confirmed at the desired value before the medium was dispensed into sterile Petri dishes. Approximately 25 mL of motility medium was dispensed into each 90-mm Petri dish, and plates were allowed to solidify before bacterial inoculation.

CBD-adapted and CBD-susceptible *S.* Typhimurium cultures were prepared for the motility experiments. A standardized inoculum of 2 µL was introduced at the center of each semi-solid agar plate. Care was taken to maintain a consistent inoculation position and depth across all plates to minimize technical variation in bacterial migration. For each bacterial strain and pH condition, three replicate plates were prepared from the same culture. These plates were technical replicates and were not treated as independent biological observations. Following inoculation, the plates were incubated at 37 °C for 60 h. Motility was assessed at 0, 24, 48, and 60 h after inoculation. At each time point, bacterial migration was quantified by measuring the diameter of the motility zone (mm) extending outward from the original inoculation site. Where migration was not perfectly circular, two perpendicular diameters were measured and averaged to obtain a representative motility diameter for each plate. Measurements obtained immediately after inoculation (0 h) served as the baseline. Motility measurements were compared descriptively between the CBD-adapted and CBD-susceptible strains at each pH and time point. pH 7.0 represented the approximately neutral condition against which the responses under pH 3.0 and pH 12.0 were compared.

### 4.6. Growth Assessment at Different pH Levels

The growth kinetics of *Salmonella* Typhimurium were evaluated in LB broth adjusted to pH 3, 5, 7, or 12. The pH was adjusted with 1 N HCl for acidic conditions or 1 N NaOH for alkaline conditions while stirring. Overnight cultures were inoculated into the broth and incubated at 37 °C with shaking. Aliquots of 200 µL were seeded in a 96-well microtiter plate (Fisherbrand, Fisher Scientific, Fair Lawn, NJ, USA) at an OD_600_ of 0.3 to represent early-log-phase cultures. Growth was monitored by measuring OD_600_ at regular intervals with a SpectraMax ABS Plus plate reader (Molecular Devices LLC, San Jose, CA, USA).

### 4.7. Nutrient Starvation Assessment

To investigate the influence of nutrient starvation, nutrient-depleted medium was supplemented with CBD at 5, 50, and 100 µg/mL, following a similar approach to that used in [16]. Both CBD-adapted and CBD-susceptible strains were inoculated into the medium and incubated at 37 °C with shaking. Changes in optical density at 600 nm (OD_600_) were monitored at regular intervals using a SpectraMax^®^ ABS Plus plate reader (Molecular Devices LLC, San Jose, CA, USA). The resulting curves were interpreted descriptively; no inferential significance claim was drawn from technical replicates.

The effects of nutrient starvation and cannabidiol (CBD) exposure on CBD-adapted and CBD-susceptible *Salmonella enterica* serovar Typhimurium strains were evaluated using 1× phosphate-buffered saline (PBS) as a nutrient-depleted medium. PBS was selected to establish a nutrient-starvation environment because it maintains physiological pH and osmotic conditions while providing no readily metabolizable carbon or nitrogen source to support bacterial proliferation. Thus, changes in optical density under these conditions were interpreted primarily in the context of bacterial persistence or survival under nutrient limitation rather than conventional growth in a nutrient-rich medium.

The 1× PBS formulation used for the nutrient-starvation experiments consisted of approximately 137 mM of NaCl, 2.7 mM of KCl, 10 mM of Na_2_HPO_4_, and 1.8 mM of KH_2_PO_4_, adjusted to approximately pH 7.4. The buffer therefore contained no added glucose or other organic carbon sources, amino acids, peptides, proteins, or other conventional nutrients required to support sustained bacterial growth. Although PBS contains inorganic phosphate as part of its buffering system, it was used here to model carbon- and nitrogen-limited conditions, rather than complete phosphorus deprivation.

CBD-adapted and CBD-susceptible *S*. Typhimurium strains were grown under their respective maintenance conditions prior to the starvation experiment. Bacterial cultures were collected via centrifugation at 12,000 rpm, washed thrice with sterile 1× PBS to minimize carryover of nutrients from the preceding growth medium, and subsequently resuspended in sterile 1× PBS. For the starvation assay, standardized bacterial suspensions in 1× PBS were exposed to CBD at final concentrations of 5, 50, and 100 µg/mL. A corresponding 0 µg/mL of CBD control containing bacteria suspended in 1× PBS without CBD was included to distinguish the effects of CBD exposure from those associated with nutrient starvation alone. Where CBD was prepared using an organic solvent, an equivalent concentration of the solvent was included in the appropriate vehicle control.

The CBD-adapted and CBD-susceptible bacterial suspensions were incubated at 37 °C under shaking at 121 rpm. Changes in bacterial suspension density were monitored at predetermined intervals by measuring the optical density at 600 nm (OD_600_) using a SpectraMax^®^ ABS Plus microplate reader (Molecular Devices LLC, San Jose, CA, USA). Measurements were obtained from six technical replicate wells per treatment condition from the same prepared bacterial suspension. These wells were not independent biological replicates. Appropriate PBS and PBS-plus-CBD blanks without bacterial cells were included to account for background absorbance associated with the buffer and CBD.

OD_600_ values were plotted as a function of incubation time to compare the responses of the CBD-adapted and CBD-susceptible strains under nutrient-starvation conditions and across CBD concentrations. Because 1× PBS does not provide the nutrients required for sustained bacterial proliferation, OD_600_ measurements obtained in this assay were used principally to monitor changes in bacterial suspension density during starvation and CBD exposure rather than being interpreted as evidence of unrestricted bacterial growth. Comparisons between strains and CBD concentrations were subsequently used to determine whether prior adaptation to CBD was associated with altered persistence under nutrient-limited conditions.

### 4.8. Quantitative Assessment of Biofilm Formation

Bacterial cultures were grown in LB broth in 96-well plates under the indicated CBD treatment and untreated-control conditions and incubated at 37 °C for 24 h or 60 h. Biofilms were stained with crystal violet, washed, and solubilized in ethanol. Absorbance at 412 nm was measured to quantify retained crystal violet.

The effects of cannabidiol (CBD) on biofilm formation by laboratory-derived CBD-adapted and CBD-susceptible *Salmonella enterica* serovar Typhimurium strains were evaluated using a crystal-violet biofilm assay. Overnight bacterial cultures were prepared in Luria–Bertani (LB) broth. For biofilm formation, 25 µL of the standardized bacterial suspension was inoculated into 100 µL of LB broth per well. CBD was added to achieve final concentrations of 5, 50, and 100 µg/mL. An untreated/vehicle control containing an equivalent volume of deionized water without CBD was included as the negative control. Hydrogen peroxide (5%) was included as a positive control for biofilm disruption. Appropriate medium-only wells without bacterial inoculation were included as blanks to determine background absorbance associated with the medium, CBD, staining reagents, and microplate.

The plates were incubated at 37 °C for 24 and 60 h under static conditions to permit biofilm development. Following incubation, the planktonic bacterial suspension was carefully removed without disturbing the surface-associated biofilm. Each well was gently washed three times with 200 µL of sterile 1× PBS to remove non-adherent and loosely attached bacterial cells. The remaining adherent biofilms were stained with 5% (*w*/*v*) crystal violet by adding 100 µL of crystal-violet solution to each well. Plates were incubated with the stain for 30 min at room temperature. Excess crystal violet was subsequently removed, and the wells were washed three times with sterile distilled water or PBS until the wash solution was visually clear. Plates were then allowed to air-dry. Biofilm-associated crystal violet was solubilized by adding 100 µL of 70% ethanol to each well and incubating for 10 min to ensure adequate extraction of the retained dye. Absorbance was measured at 412 nm using a SpectraMax^®^ ABS Plus microplate reader (Molecular Devices LLC, San Jose, CA, USA). For each condition, three wells from the same experimental preparation were analyzed as technical replicates. They were not independent biological replicates. Raw absorbance values were corrected for background by subtracting the mean absorbance of the corresponding bacteria-free blank wells: Corrected biofilm absorbance = OD(sample) − OD(blank).

Blank-corrected absorbance values were used for descriptive comparisons between the CBD-adapted and CBD-susceptible strains and among CBD treatment concentrations. Higher crystal-violet absorbance was interpreted as greater surface-associated biofilm biomass, whereas lower absorbance indicated reduced biofilm biomass. Because crystal-violet staining measures total attached biomass rather than bacterial viability specifically, the results were interpreted as changes in overall biofilm biomass and not as a direct measure of the number of viable cells.

### 4.9. SEM Analysis of Biofilm Formation

Scanning electron microscopy (SEM) was subsequently used to provide complementary morphological assessment of biofilm architecture following CBD treatment. CBD-adapted and CBD-susceptible *S*. Typhimurium strains were cultured in sterile six-well plates (Fisherbrand, Fisher Scientific, Fair Lawn, NJ, USA) containing LB broth. The bacterial inoculum was standardized to an OD_600_ of 0.60, and 100 µL was added to each well containing 90 µL. Biofilms were exposed to CBD at final concentrations of 5 or 100 µg/mL. An untreated/vehicle group containing the equivalent volume of deionized water served as the negative control, whereas 5% hydrogen peroxide served as the positive control for oxidative biofilm disruption. Samples were incubated at 37 °C for 24 and 48 h under static conditions to permit biofilm formation and treatment.

Following incubation, the culture medium and non-adherent cells were carefully removed. The adherent biofilms were gently washed three times with sterile 1× PBS to remove residual planktonic cells without disrupting the attached biofilm structure. Biofilms were subsequently fixed with 10% formaldehyde for 10 min at room temperature. Following fixation, specimens were washed three times with 1× PBS and dehydrated sequentially using increasing concentrations of ethanol (50%, 70%, and 90%). Samples were then vacuum-dried to remove residual moisture while preserving the biofilm-associated structures as much as possible.

Dried specimens were sputter-coated with a thin layer of gold using an EMS 150R ES ion-sputtering system and examined using a JEOL JSM-6010LA analytical scanning electron microscope (JEOL Ltd., Tokyo, Japan) with InTouchScope™ software. Images were acquired at 10 kV and at 1000×, 5000×, and 10,000× magnifications. Identical imaging parameters and comparable regions of the substratum were used across experimental groups to facilitate morphological comparison.

SEM images were evaluated for treatment-associated differences in bacterial surface attachment, cellular aggregation, biofilm coverage, and overall biofilm architecture between CBD-adapted and CBD-susceptible *S.* Typhimurium. SEM was used as a qualitative morphological complement to the quantitative crystal-violet assay rather than as a direct measure of viable bacterial numbers.

### 4.10. Statistical Analysis

Two *Salmonella* Typhimurium strains—the laboratory-derived CBD-adapted derivative and its CBD-susceptible parental strain—were examined. The repeated wells, plates, and qPCR measurements were technical replicates and were not independent biological observations. Technical replicates were averaged or summarized only to describe within-assay variation. Because no independent biological replicates were included, neither parametric nor nonparametric inferential testing can support generalization beyond these two strains. No *p*-values or inferential significance symbols are presented in the revised figures. The results are presented descriptively, and confirmation will require independent biological cultures, additional isolates, and a statistical analysis based on the correct experimental unit.

## 5. Conclusions

The *flgA* gene functions within the coordinated flagellar-assembly program. It encodes the periplasmic chaperone FlgA, which assists assembly of the FlgI P ring; FlgA is not itself the structural P-ring protein.

In this study, we observed an association between prolonged CBD exposure, higher *flgA* mRNA expression, and altered phenotypes in a laboratory-derived CBD-adapted *Salmonella* Typhimurium strain. The adapted strain showed greater motility at pH 7, but the response was not uniform across all pH conditions. Both strains showed reduced relative OD_600_ at pH 3 and pH 12, and both declined under nutrient starvation. Biofilm-associated measurements also varied with strain, CBD concentration, and incubation time. Because *flgA* was not manipulated, we cannot conclude that its higher expression caused the motility, biofilm, or stress-response patterns. Likewise, the study did not measure invasion, immune evasion, cellular ATP, proton motive force, CBD efflux, CBD utilization, or viability during the optical-density experiments. Our findings therefore provide an exploratory framework for future studies using independently derived CBD-adapted lineages, MIC/MBC and stability testing, direct viability measurements, and *flgA* knockout, complementation, or controlled overexpression experiments.

## Figures and Tables

**Figure 1:**
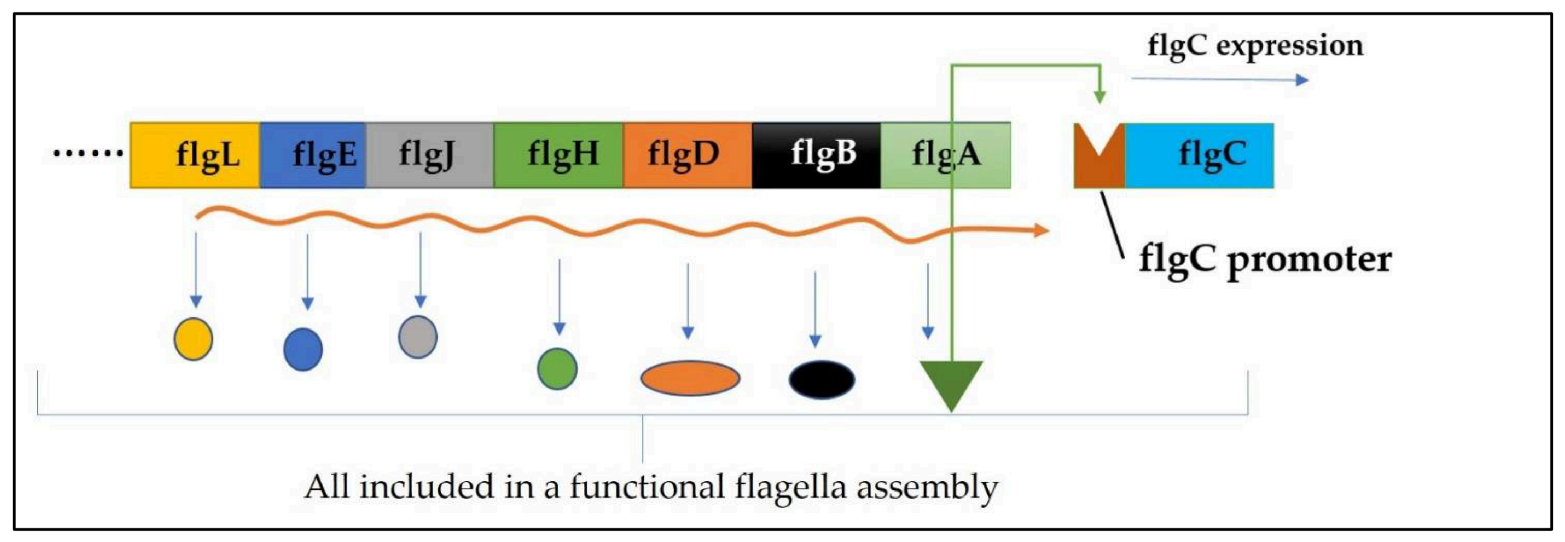
Diagrammatic overview of genes involved in flagellar biosynthesis. Coordinated expression of the illustrated genes contributes to functional flagellar assembly. FlgA supports assembly of the FlgI P-ring protein; it is not a transcription factor.

**Figure 2:**
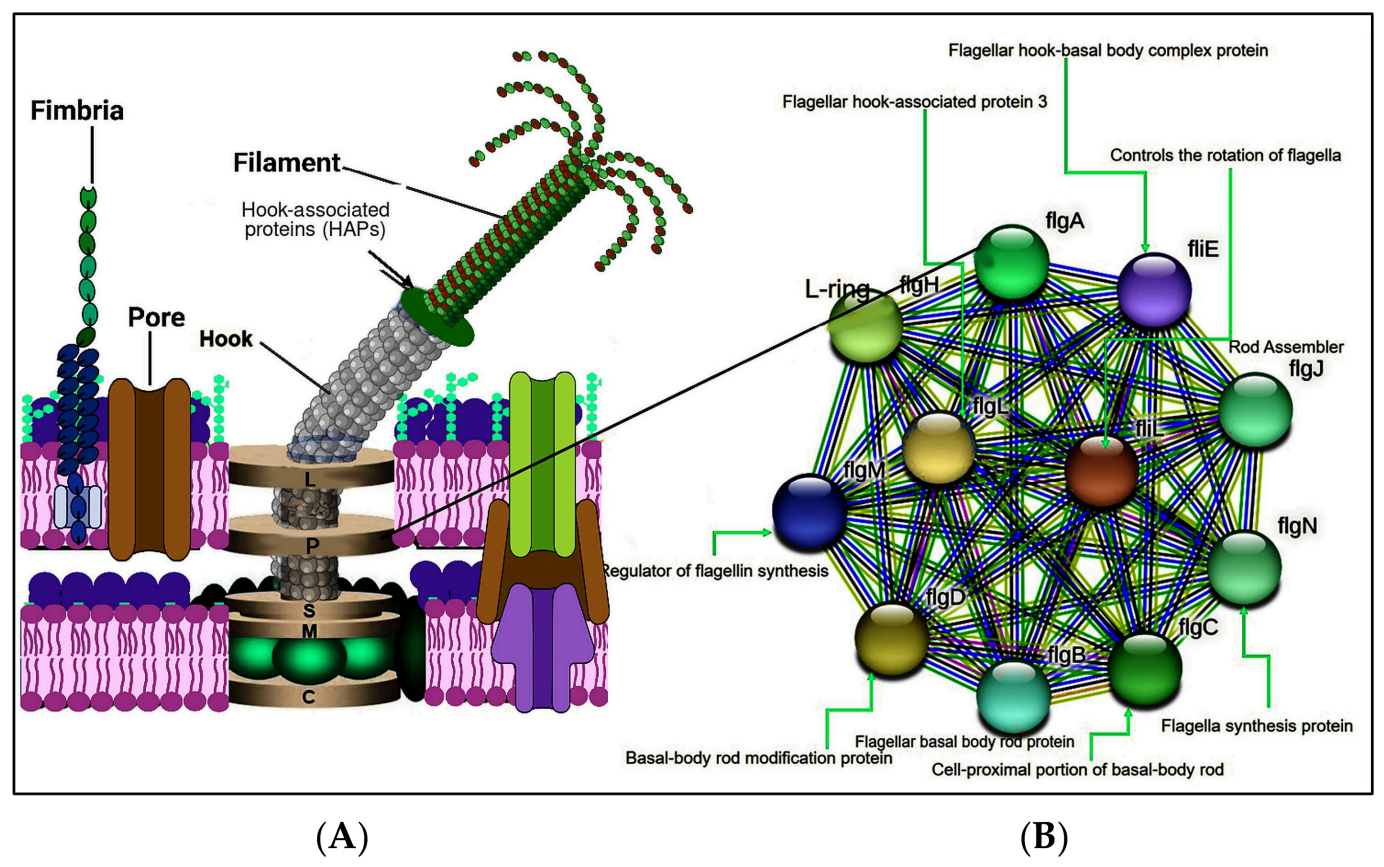
(**A**) Major structural components of the bacterial flagellum and associated surface structures. (**B**) STRING protein-interaction network centered on FlgA. Network edges indicate predicted or documented functional associations and do not by themselves establish direct physical interaction. In panel (**A**), L and P label the outer-membrane and peptidoglycan rings, respectively; M and S denote the membrane and supramembrane components of the MS ring; and C labels the cytoplasmic ring.

**Figure 3:**
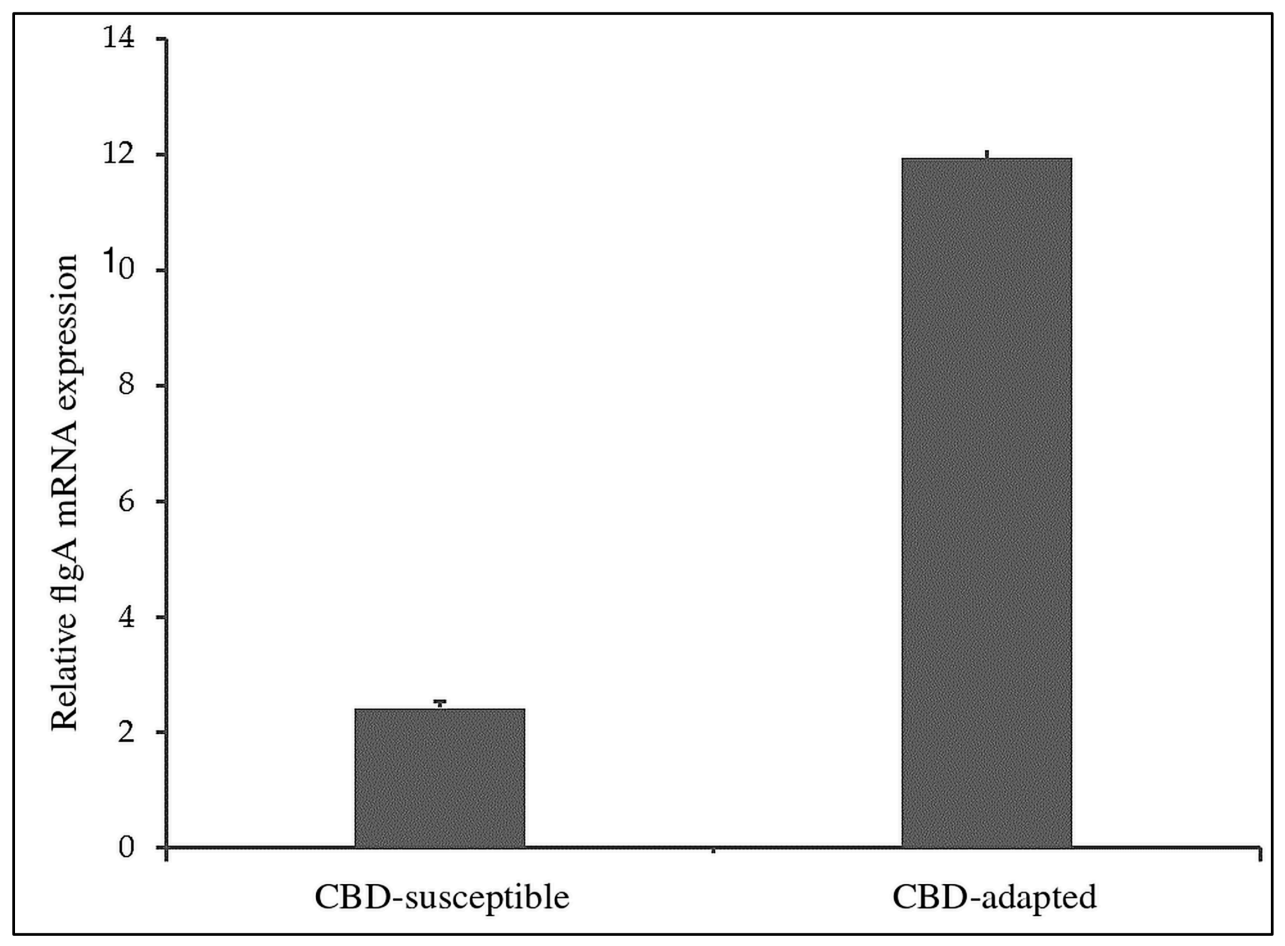
Relative *flgA* mRNA expression in the laboratory-derived CBD-adapted and CBD-susceptible *Salmonella* Typhimurium strains. Bars and error bars summarize technical qPCR measurements descriptively. No inferential significance comparison is presented because independent biological replicates were not included.

**Figure 4:**
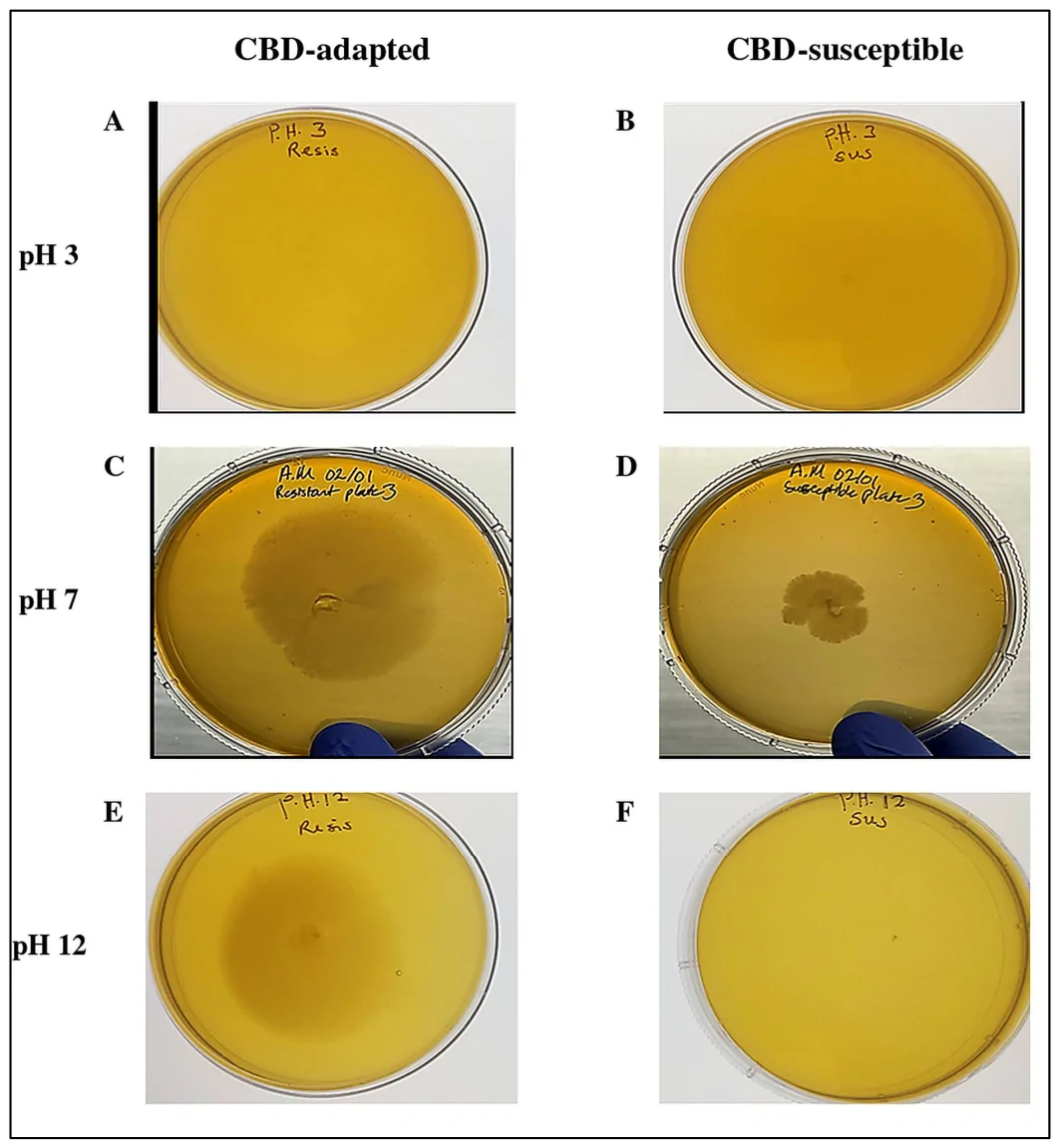
Motility plates for the laboratory-derived CBD-adapted and CBD-susceptible *Salmonella* Typhimurium strains. Panels show the CBD-adapted and CBD-susceptible strains at pH 3 (**A**,**B**), pH 7 (**C**,**D**), and pH 12 (**E**,**F**), respectively.

**Figure 5:**
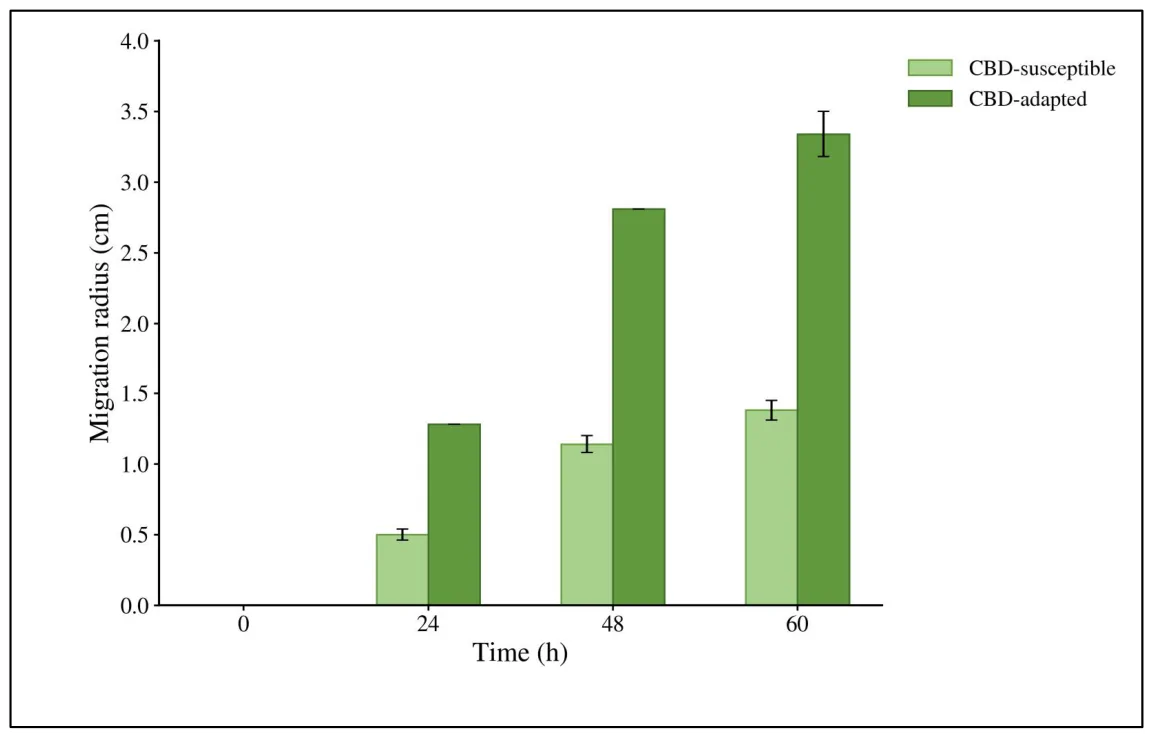
Migration radius of the laboratory-derived CBD-adapted and CBD-susceptible *Salmonella* Typhimurium strains on pH 7 motility agar at 0, 24, 48, and 60 h. Bars and error bars summarize technical replicate plates descriptively. No inferential significance symbols are presented.

**Figure 6:**
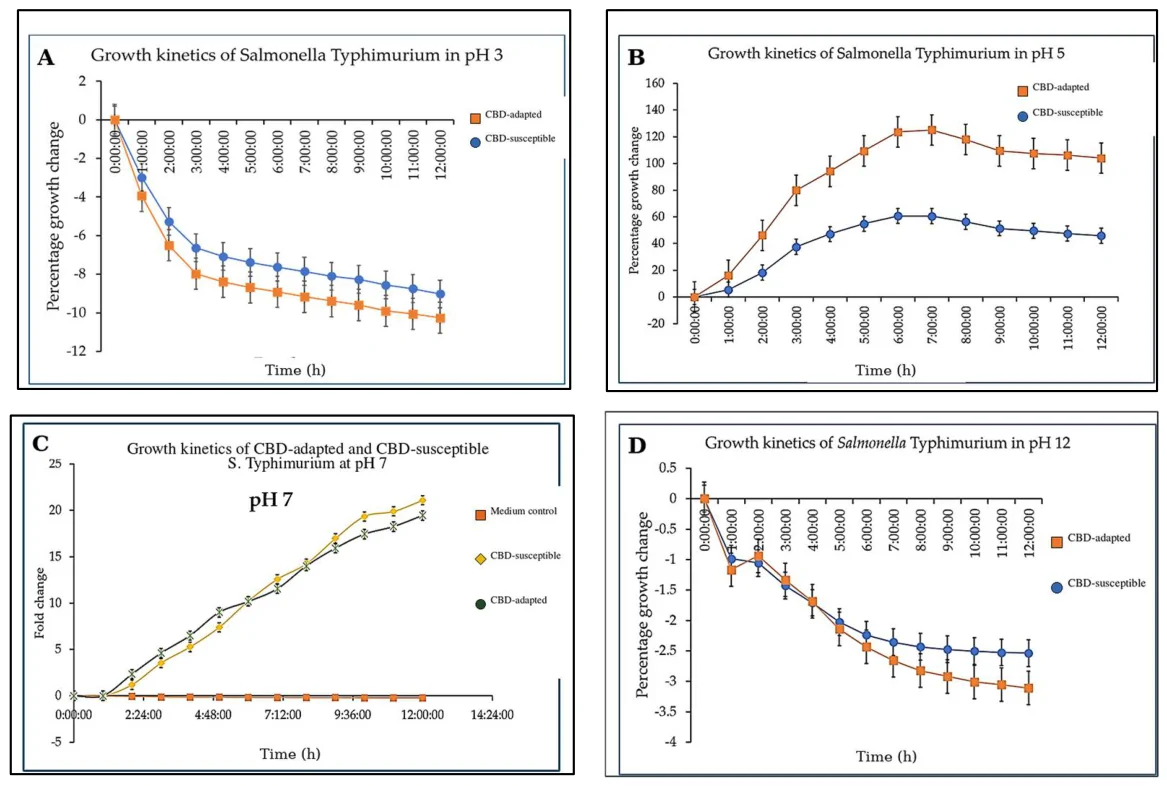
Growth assessment of laboratory-derived CBD-adapted and CBD-susceptible *Salmonella* Typhimurium from low to high pH: (**A**) pH 3, (**B**) pH 5, (**C**) pH 7, and (**D**) pH 12. Panels (**A**,**B**,**D**) show percentage change in OD_600_, whereas panel (**C**) shows fold change in OD_600_. Error bars describe technical measurement variation. In panel (**D**), the x-axis represents hourly measurements from 0 to 12 h.

**Figure 7:**
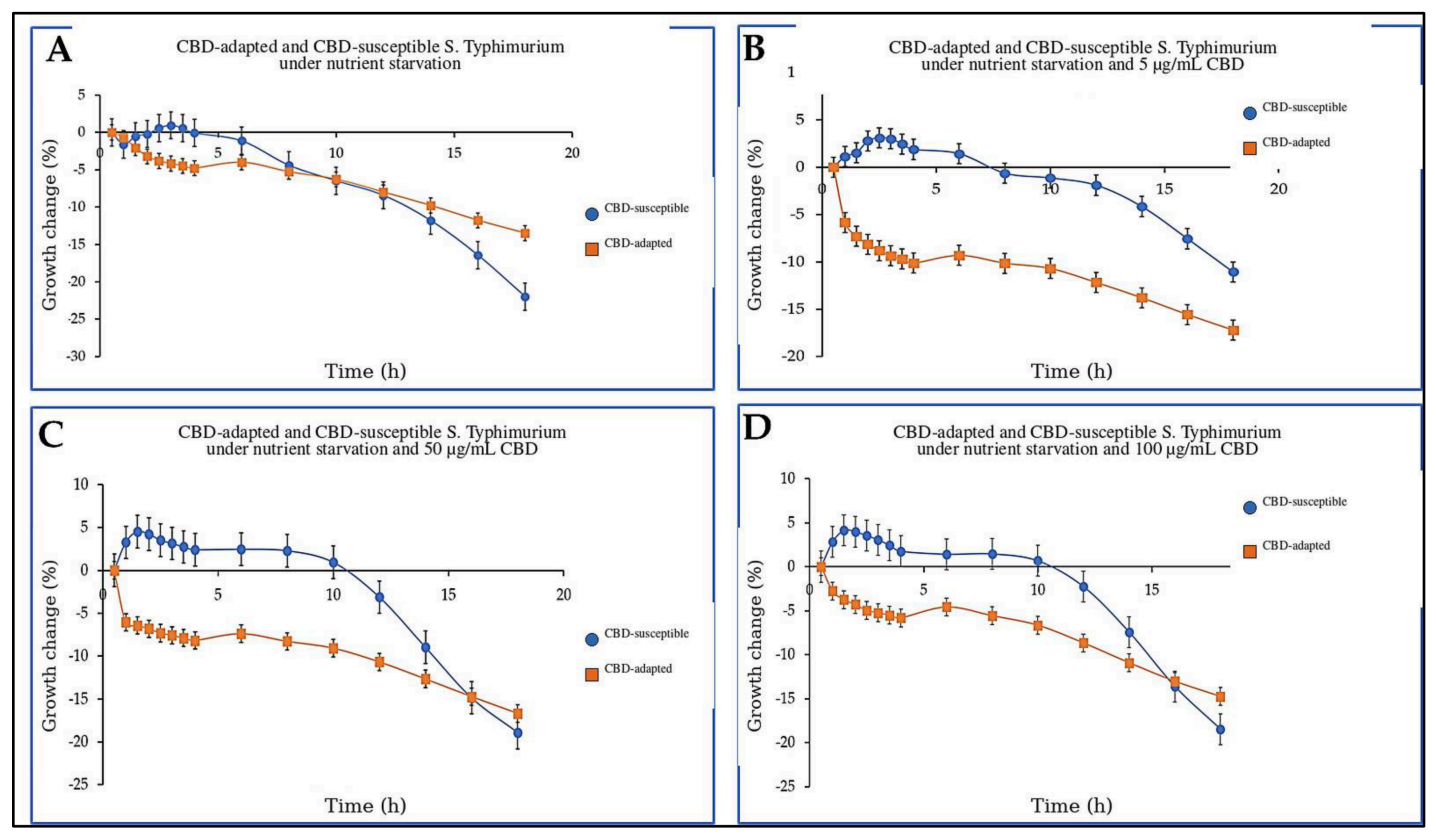
Nutrient-starvation assessment of laboratory-derived CBD-adapted and CBD-susceptible *Salmonella* Typhimurium in PBS containing (**A**) 0, (**B**) 5, (**C**) 50, or (**D**) 100 µg/mL of CBD. The y-axes show relative change in OD_600_, and the x-axes show time in hours.

**Figure 8:**
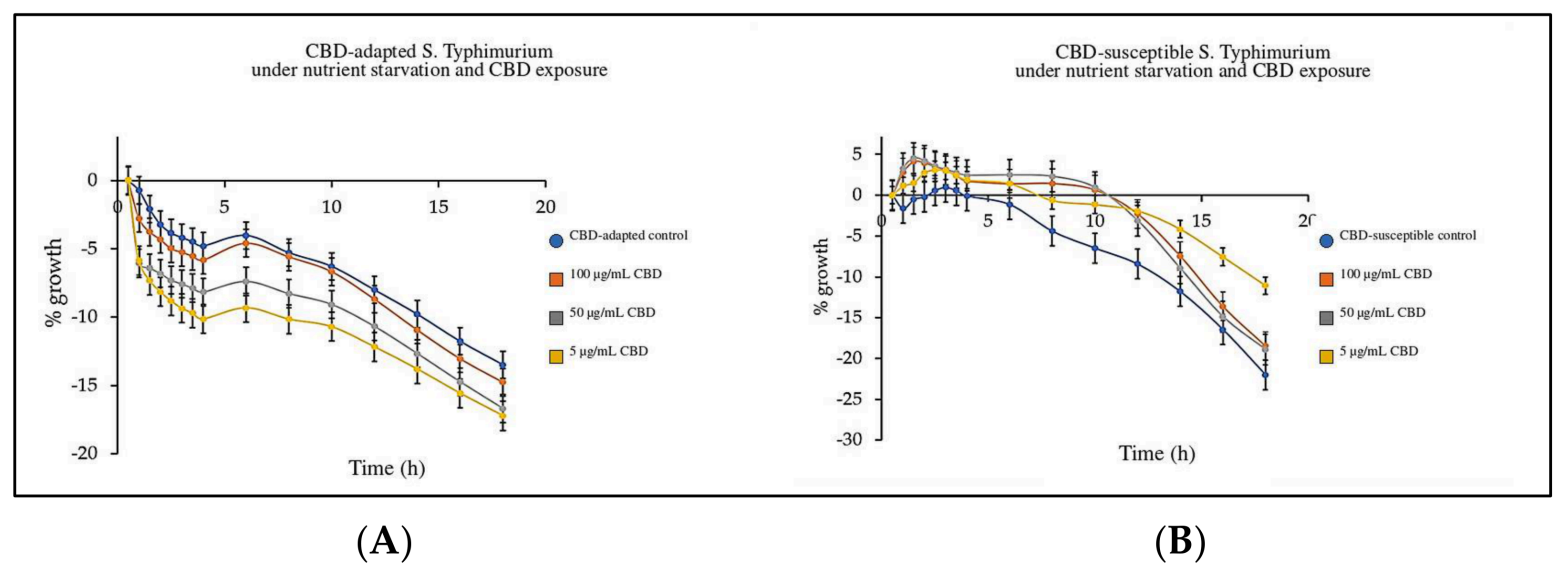
Strain-specific growth kinetics under nutrient starvation and CBD exposure: (**A**) laboratory-derived CBD-adapted *Salmonella* Typhimurium and (**B**) the CBD-susceptible parental strain. Curves show the conditions with 0, 5, 50, and 100 µg/mL of CBD.

**Figure 9:**
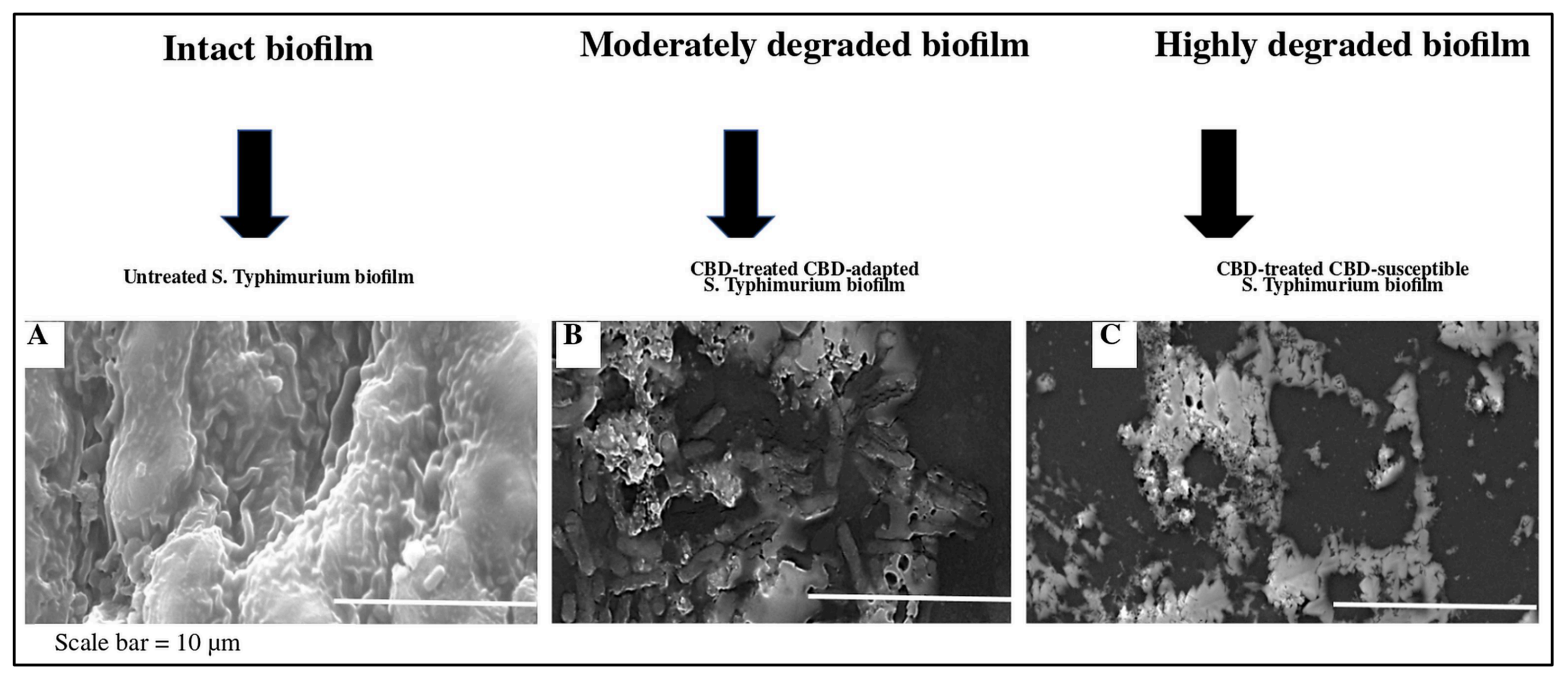
Representative SEM images of *Salmonella* Typhimurium biofilms: (**A**) untreated intact biofilm, (**B**) CBD-treated biofilm formed by the CBD-adapted strain, and (**C**) CBD-treated biofilm formed by the CBD-susceptible strain. The images provide qualitative morphological comparisons under the indicated conditions. Scale bar = 10 µm.

**Figure 10:**
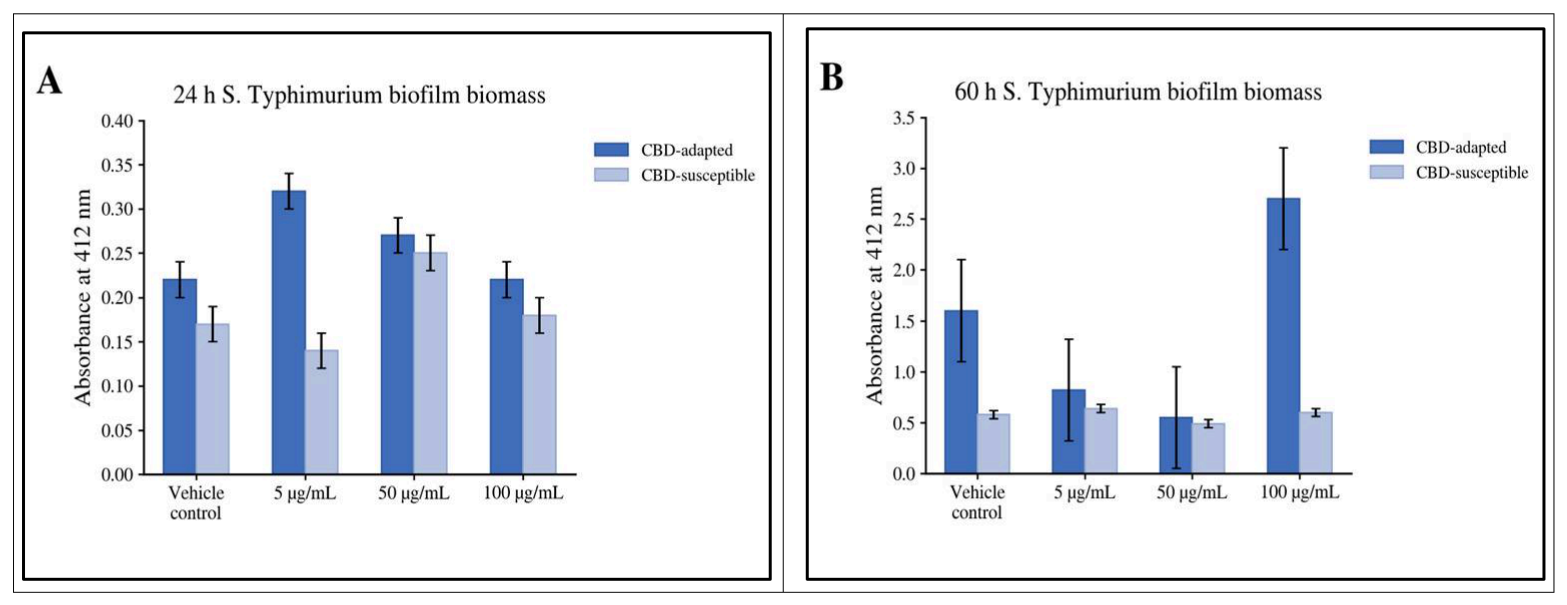
Crystal-violet quantification of biofilm biomass produced by the laboratory-derived CBD-adapted and CBD-susceptible *Salmonella* Typhimurium strains after (**A**) 24 h and (**B**) 60 h. Bars and error bars summarize technical replicate wells descriptively. No inferential significance symbols are presented.

**Figure 11:**
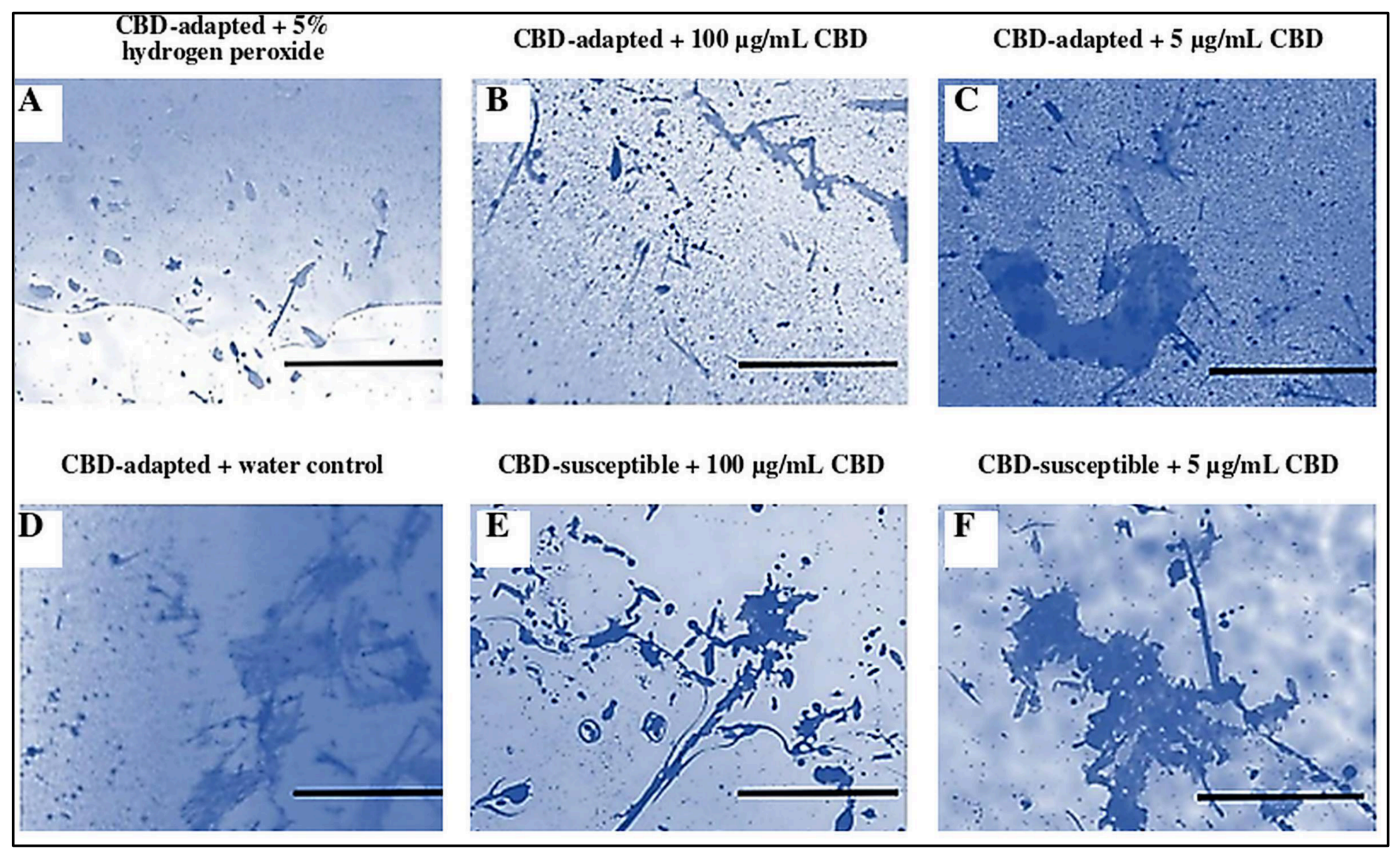
Microphotographs of crystal-violet-stained *Salmonella* Typhimurium biofilms under the displayed treatments: (**A**) CBD-adapted strain with 5% H_2_O_2_, (**B**) CBD-adapted strain with 100 µg/mL of CBD, (**C**) CBD-adapted strain with 5 µg/mL of CBD, (**D**) CBD-adapted strain water control, (**E**) CBD-susceptible strain with 100 µg/mL of CBD, and (**F**) CBD-susceptible strain with 5 µg/mL of CBD. Scale bar = 100 µm.

**Table 1:** Endpoint motility of the two *Salmonella* Typhimurium strains at pH 3 and pH 12 after 48 h. The pH 7 experiment was collected as a time course and is presented separately in Figure 5 rather than repeated as a single endpoint in this table. Values are the originally reported means ± dispersion from technical replicate plates and are presented descriptively; they are not inferential estimates.

pH	CBD-Susceptible	CBD-Adapted
	Average migration radius after 48 h (cm)	
3	0.0 ± 0.00	0.0 ± 0.00
12	0.0 ± 0.00	1.6 ± 2.26

## Data Availability

The original contributions presented in this study are included in the article. Further inquiries can be directed to the corresponding authors.
